# Ambient Air Pollution and Kawasaki Disease: A Systematic Review and Meta‐Analysis of Epidemiological Evidence

**DOI:** 10.1002/puh2.70448

**Published:** 2026-11-07

**Authors:** Amogh Verma, Ranjana Sah, Pratima Pandey, Asif Jan, Daniel Dubwa

**Affiliations:** ^1^ Department of Internal Medicine Rama Medical College Hospital and Research Centre Hapur Uttar Pradesh India; ^2^ Dr. D. Y. Patil Medical College Hospital and Research Centre Dr. D. Y. Patil Vidyapeeth (Deemed‐to‐be‐University) Pune Maharashtra India; ^3^ Department of Pharmacy Practice National Institute of Pharmaceutical Education and Research S.A.S Nagar Punjab India; ^4^ Department of Pharmacy University of Peshawar Peshawar Khyber Pakhtunkhwa Pakistan; ^5^ Saidu Group of Teaching Hospitals Saidu Sharif, Swat Khyber Pakhtunkhwa Pakistan; ^6^ District Headquarter Hospital Charsadda Charsadda Khyber Pakhtunkhwa Pakistan; ^7^ Department of Pharmacy Makerere University Kampala Uganda

**Keywords:** air pollution, Kawasaki disease, ozone, particulate matter, PM_2.5_

## Abstract

**Background:**

Kawasaki disease (KD) is a childhood vasculitis with a multifactorial etiology involving genetic susceptibility and environmental influences. Ambient air pollution has been proposed as a potential environmental trigger, but epidemiological findings have been inconsistent across pollutants, populations, and exposure periods. This systematic review and meta‐analysis aimed to synthesize the association between ambient air pollution and KD.

**Methods:**

PubMed, Embase, and Web of Science were searched from database inception through December 29, 2024, for observational studies evaluating associations between ambient air pollutants and KD. Multivariable‐adjusted estimates were preferentially used for quantitative synthesis. Hazard ratios and odds ratios were interpreted as relative risks, and estimates were standardized to a 10 µg/m^3^ increase in pollutant concentration where applicable. Random‐effects models were used to obtain pooled relative risks, with heterogeneity assessed using *I* ^2^ statistics.

**Results:**

Thirteen studies met the inclusion criteria. The studies included diverse epidemiological designs and were predominantly conducted in Asian populations, with additional studies from North America and Italy. Across the quantitative synthesis, no statistically significant association was observed between a standardized 10 µg/m^3^ increase in individual pollutants and KD occurrence. The pooled relative risks were 1.00 (95% confidence interval [CI], 0.985–1.016) for PM_2.5_, 1.00 (95% CI, 0.983–1.022) for PM_10_, 1.008 (95% CI, 0.966–1.051) for SO_2_, 0.99 (95% CI, 0.989–1.009) for NO_2_, and 1.013 (95% CI, 0.981–1.046) for O_3_ and CO. For PM_2.5_, between‐study heterogeneity was moderate (*I* ^2^ = 43.8%), whereas Egger's test showed no evidence of significant publication bias (*p* = 0.2703).

**Conclusion:**

Current evidence does not demonstrate a statistically significant overall association between ambient air pollution and KD. However, associations may vary by pollutant, exposure timing, geographical setting, and meteorological conditions. Future studies should use standardized exposure windows and account for key meteorological factors to clarify these potential context‐specific associations.

## Graphical abstract

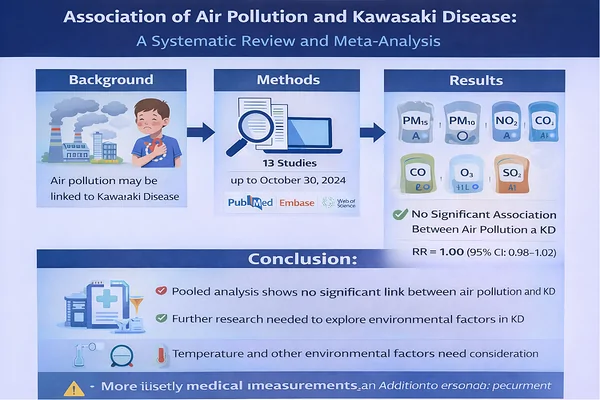

## 1 Introduction

Kawasaki disease (KD) is a systemic vasculitis primarily involving medium‐sized arteries and occurs predominantly in children younger than 5 years of age. It is recognized as the leading cause of acquired heart disease in children in developed countries [1]. The development of KD is considered multifactorial, involving interactions between genetic susceptibility and environmental influences [2]. Despite considerable research into its underlying mechanisms, the specific factors responsible for initiating the disease remain incompletely understood. Emerging evidence indicates that environmental and geographical factors, including variations in weather conditions and seasonal patterns, may contribute to the timing and occurrence of KD [3, 4, 5]. In particular, climatological observations from different regions have led to the hypothesis that airborne agents, including wind‐borne antigens or toxins, may serve as potential environmental triggers [6, 7].

Air pollution has emerged as an important environmental factor of interest because of its potential to promote systemic inflammation and contribute to immune‐mediated disease processes [8, 9]. Epidemiological evidence has linked short‐term exposure to fine particulate matter (PM_2.5_) with systemic inflammatory responses and adverse cardiovascular outcomes, including coronary atherosclerosis and ischemic coronary events [10, 11]. Children may be especially susceptible to the harmful effects of ambient air pollution because of their developing respiratory and immune systems and their relatively greater pollutant exposure per unit of body weight [12].

Experimental and epidemiological evidence further suggests that short‐term exposure to ambient PM_2.5_ can stimulate systemic and proinflammatory responses in children [13]. Such inflammatory processes may be relevant to KD pathogenesis, in which immune activation, endothelial injury, oxidative stress, and inflammatory responses play important roles [14]. Findings from experimental models, including murine models of KD, together with transcriptomic investigations of blood samples from children with KD, have demonstrated activation of innate immune pathways and inflammatory responses [15, 16]. Fine particulate matter may contribute to these processes through toll‐like receptor‐mediated mechanisms and inflammasome activation [17, 18]. In addition, epidemiological studies have suggested that exposure to ambient air pollution during pregnancy and early life may be associated with subsequent development of KD in children [19].

Although several epidemiological studies have investigated the potential relationship between ambient air pollution and the occurrence of KD, their findings have been inconsistent [19, 20, 21]. Therefore, this systematic review aimed to synthesize the available epidemiological evidence regarding the association between air pollution exposure and KD onset. By evaluating pollutant‐specific associations across available studies, we sought to clarify the potential contribution of air pollution to the development of KD and identify important gaps requiring further investigation.

We hypothesized that the association between air pollution and KD may be modified or confounded by seasonally varying meteorological conditions, including temperature, humidity, and wind patterns, which may influence both pollutant concentrations and KD incidence.

## 2 Methods

### 2.1 Study Design and Reporting Framework

We undertook a systematic review and meta‐analysis to determine whether exposure to ambient air pollutants is associated with the occurrence of KD. The review methodology was developed and reported in accordance with the Preferred Reporting Items for Systematic Reviews and Meta‐Analyses (PRISMA) recommendations (Table S1) [21]. The protocol of the study was not preregistered.

### 2.2 Literature Identification

Relevant literature was identified through systematic searches of three electronic databases: PubMed, Embase, and Web of Science. Records published from database inception through December 29, 2024, were considered. The search strategy combined terminology describing air pollution and individual pollutants with terms referring to KD. Pollutant‐related terms included PM_2.5_, PM_10_, particulate matter, nitrogen oxides, nitrogen dioxide (NO_2_), sulfur dioxide (SO_2_), ozone (O_3_), carbon monoxide (CO), black carbon, and general terms, such as air pollution and pollutant. Disease‐related terms included KD, Kawasaki syndrome, and mucocutaneous lymph node syndrome. The complete database‐specific search strategies are provided in Table S2.

### 2.3 Selection of Eligible Studies

Studies were considered for inclusion when they investigated the relationship between air‐pollution exposure and KD using an observational or epidemiological study design, including cohort, case–control, or cross‐sectional approaches. Eligible studies were required to provide a clearly defined assessment of pollutant exposure and a reproducible definition or ascertainment method for KD. KD ascertainment could be based on physician diagnosis, relevant ICD‐9 or ICD‐10 diagnostic codes, or IVIG treatment records, as reported by the individual study. Studies were also required to provide an effect estimate with a corresponding 95% confidence interval (CI), or sufficient numerical information to derive these measures.

We excluded review articles, commentaries, and studies based exclusively on animal or experimental models.

### 2.4 Screening Procedure

All records retrieved from the electronic searches were screened independently by two reviewers. Screening was initially performed at the title and abstract level, followed by examination of the full text of potentially eligible articles. Differences in eligibility assessment were resolved through discussion between the reviewers; when consensus could not be reached, a third reviewer was consulted. Rayyan was used to facilitate the screening and study‐management process. A detailed account of study identification, screening, eligibility assessment, and final inclusion is provided in the PRISMA flow diagram (Figure 1).

**FIGURE 1:**
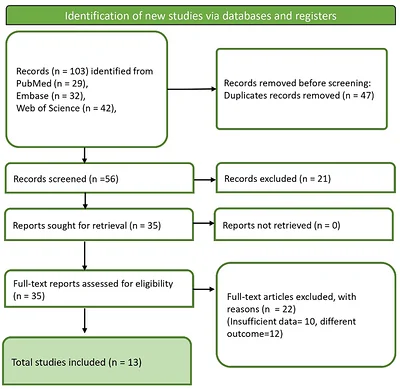
PRISMA flowchart depicting study selection process.

### 2.5 Data Collection and Methodological Appraisal

A predefined data‐extraction framework was used to collect information from each eligible study. Extracted variables included publication details, geographical location, study design, sample characteristics, participant age and sex, criteria used to identify KD, methods used to characterize air‐pollution exposure, pollutant concentrations or exposure contrasts, and reported measures of association. Particular attention was given to multivariable‐adjusted estimates and their corresponding 95% CIs, which were preferentially considered for quantitative synthesis.

Two reviewers independently extracted the study information. Any discrepancies were reviewed and resolved by discussion, with further adjudication when required. The methodological quality of observational studies was assessed using the Newcastle–Ottawa scale (NOS), with the resulting assessments presented in Table S3.

### 2.6 Data Synthesis and Statistical Analysis

#### 2.6.1 Statistical Analysis

All statistical analyses were performed using R software (version 4.4). Because differences in study populations, exposure assessment, and study design were anticipated, pooled associations were estimated using a random‐effects meta‐analysis model. For studies reporting HRs, these estimates were considered comparable to RRs, whereas ORs were also interpreted as RRs on the basis of the low prevalence of KD.

For quantitative synthesis, effect estimates were converted to a common exposure scale corresponding to a 10 µg/m^3^ increase in pollutant concentration. Where studies reported estimates using different exposure increments, the reported measures were transformed to this common scale using previously published conversion methods. The analyses assumed a linear relationship between pollutant concentration and KD risk, consistent with approaches adopted in previous epidemiological investigations. Pooled associations were expressed as RRs with corresponding 95% CIs.

Between‐study variability was quantified using the *I* ^2^ statistic, with values of approximately 25%, 50%, and 75% considered indicative of low, moderate, and high heterogeneity, respectively. The stability of the pooled estimates was evaluated using leave‐one‐out sensitivity analyses, in which each study was sequentially removed and the meta‐analysis repeated. A two‐sided *p* value <0.05 was considered statistically significant. Pollutant‐specific analyses were conducted separately for each pollutant to estimate the association between individual pollutant exposures and KD risk.

## 3 Results

### 3.1 Literature Search

A total of 90 records were identified through database searches: 24 from Embase, 27 from PubMed, and 39 from Web of Science. After removing 42 duplicates, 48 unique records remained for screening. Of these, 19 were excluded on the basis of titles and abstracts for not meeting inclusion criteria, leaving 29 full‐text articles for detailed evaluation. Following further analysis, 16 publications were excluded for reasons such as being irrelevant [10] or conference abstracts [6]. Ultimately, 13 studies [19, 20, 21, 22, 23, 24, 25, 26, 27, 28, 29, 30, 31] were included in the systematic review.

### 3.2 Characteristics of Included Studies

The characteristics of the included studies are presented in Table 1. Air pollutant exposure was estimated using methods such as land‐use regression [22], monitoring stations [19, 20, 21, 23, 25, 27, 28, 30, 31], or inverse distance weighting [25], and other methods [21, 22, 26, 29, 30]. These studies were conducted across several countries, with the majority (six studies) from Asia, including South Korea (three studies), Japan (three studies), China [2], and Taiwan (two studies). Other countries represented were the United States, Canada, and Italy. Study designs varied, with the most common being case‐crossover (five studies), followed by longitudinal (three studies), retrospective cohort (two studies), and one each of nested case–control, ecological, and time‐series studies. Sample sizes ranged from 185 to 505,336 participants, with varying proportions of male participants. Most studies focused on children under 5 years old, especially those with KD.

**TABLE 1:** Characteristics of included studies.

Study	Country	Study design	Sample size (male, %)	Age	Population
Zeft et al. [20]	The United States, Canada	Case‐crossover	3009 (NA)	NA	Children with KD
Kwon et al. [28]	South Korea	Case‐crossover	51,486 (58.2)	≥59 months	Children with KD
Yorifuji et al. [19]	Japan	Longitudinal survey	22,358 (51.7)	≥30 months	Children aged 6–30 months
Oh et al. [21]	South Korea	Case‐crossover	771 (NA)	<10 years	Hospitalized children with KD
Jung et al. [25]	Taiwan	Case‐crossover	695 (56.83)	2.54 years	Children admitted with KD
Corinaldesi et al. [23]	Italy	Ecological study	516 (58.0)	34 months	Children younger than 5 years
Lin et al. [29]	China	Time‐series analysis	2344 (69.5)	2.35 years	Hospitalized children with KD
Kuo et al. [27]	Taiwan	Nested case–control study	20,960 (54.09)	<6 years	Children younger than 6 years
Kim et al. [26]	South Korea	Longitudinal study	134,634 (51.6)	<5 years	Children younger than 5 years
Zhu et al. [31]	China	Case‐crossover study	1808 (NA)	<5 years	Children with KD
Buteau et al. [22]	Canada	Population‐based cohort study	505,336 (51.4)	3 years	Children born in Quebec
Fujii et al. [24]	Japan	Retrospective cohort study	185 (53.5)	2.1 years	Children with KD
Yoneda et al. [30]	Japan	Retrospective analysis	69,312 (NA)	<5 years	Children younger than 5 years

Abbreviation: KD, Kawasaki disease.

### 3.3 Key Findings From Studies on Air Pollution and KD

Several studies explored the relationship between exposure to air pollutants and the incidence of KD, with varying results across different geographical regions and methodologies.

A nationwide longitudinal study by Yorifuji et al. in Japan assessed both prenatal and postnatal exposure to suspended particulate matter (PM) [19]. Their findings revealed a significant association between prenatal exposure to higher levels of particulate matter (PM ≥25 µg/m^3^) and an increased risk of KD hospitalization in early childhood [19]. The odds ratio for this association was 1.59 (95% CI: 1.06–2.38) after adjusting for both individual and area‐level factors, suggesting that prenatal exposure to air pollution may increase the susceptibility to KD in later years [19].

Similarly, Fujii et al., in their investigation using data from a Japanese cohort and overdispersed generalized additive models, found a compelling link between higher concentrations of nitric oxide (NO) and sulfur dioxide (SO_2_) and an increased incidence of KD [24]. The study also highlighted a link between KD incidence and preceding hot temperatures, emphasizing the role of climate conditions in influencing KD risk [24].

In contrast, Lin et al., using time‐series analysis in Shanghai, China, did not find significant associations between air pollutants and KD incidence [29]. However, their findings did suggest that high temperatures might elevate KD risk, with a particularly interesting “J‐shaped” relationship between daily temperature fluctuations and KD incidence [29].

In Europe, Corinaldesi et al. conducted a long‐term study spanning 17 years in Emilia‐Romagna, Italy, to examine the timing of KD onset in relation to environmental conditions [23]. Although they did not identify a direct link between air pollution and KD, they found that KD cases predominantly occurred during late autumn and early spring [23]. Furthermore, their analysis suggested that specific climatic factors, such as local wind regimes, influenced the frequency and timing of KD outbreaks [23]. This points to the possibility that airborne agents, modulated by weather patterns, may play a role of trigger in KD's onset [23].

Zhu et al., in a study spanning East China from 2013 to 2020, uncovered a significant association between short‐term exposure to ozone (O_3_) and the onset of KD [31]. Specifically, each interquartile range (52.32 µg/m^3^) increase in O_3_ concentration on the day of KD onset was linked to a 16.2% (95% CI: 3.6%, 30.3%) higher risk of the disease [31]. However, this effect weakened on subsequent days, as exposures to O_3_ on lag days (1 and 2) were not statistically significant, suggesting that the immediate effects of ozone exposure are most influential in triggering KD [31]. Table 2 details exposure measurements, estimates, and effect sizes.

**TABLE 2:** Exposure characteristics and adjusted associations between air pollutants and Kawasaki disease (KD).

Study	Pollutant(s)	Exposure assessment	KD ascertainment	Exposure metric/concentration	Fully adjusted association
Zeft et al. [20]	PM_2.5_	Air‐quality monitoring stations	Physician‐operated KD registries	Range: 7.0—17.2 µg/m^3^	OR per 10 µg/m^3^ increase: 0.995 (95% CI, 0.868–1.141) for 14‐day moving average; 0.980 (95% CI, 0.915–1.050) for 2‐day moving average
Kwon et al. [28]	PM_10_, PM_2.5_, NO_2_, SO_2_, CO, O_3_	Air‐quality monitoring stations	ICD‐10 code M30.3 and IVIG treatment	IQR: PM_10_ 28.68 µg/m^3^; PM_2.5_ 14.67 µg/m^3^; NO_2_ 17.42 ppb; SO_2_ 2.79 ppb; CO 0.32 ppm; O_3_ 25.38 ppb	OR per IQR increase: PM_10_ 0.994 (0.973–1.016); PM_2.5_ 1.001 (0.978–1.025); NO_2_ 0.995 (0.962–1.028); SO_2_ 1.003 (0.972–1.035); CO 1.010 (0.981–1.040); O_3_ 1.001 (0.960–1.043)
Yorifuji et al. [19]	Suspended particulate matter (PM ≤7 µm)	Air‐quality monitoring stations	Hospital admission for KD	Categories: <20, 20–25, and ≥25 µg/m^3^	Prenatal exposure: OR per 10 µg/m^3^ increase 1.13 (0.79–1.61); OR for ≥25 µg/m^3^ 1.59 (1.06–2.38). Postnatal exposure: OR per 10 µg/m^3^ increase 1.11 (0.73–1.68); OR for ≥25 µg/m^3^ 1.41 (0.82–2.41)
Oh et al. [21]	PM_10_, SO_2_, NO_2_, CO, O_3_	CMAQ modeling data	Hospitalized patients with ICD‐10 M30.3	IQR: PM_10_ 30.847 µg/m^3^; SO_2_ 0.003 ppm; NO_2_ 0.019 ppm; CO 0.275 ppm; O_3_ 0.017 ppm	OR for PM_2.5_ per 10‐unit increase: 0.93 (0.82–1.05), 0–14‐day lag
Jung et al. [25]	CO, NO_2_, O_3_, PM_10_, SO_2_	Air‐quality monitoring stations with inverse‐distance weighting	ICD‐9‐CM code assigned by a pediatrician	IQR: CO 0.34 ppm; NO_2_ 13.34 ppb; O_3_ 28.73 ppb; PM_10_ 40.60 µg/m^3^; SO_2_ 3.47 ppb	OR per IQR increase: O_3_ 1.21 (1.01–1.44); NO_2_ 1.08 (0.89–1.30); CO 1.01 (0.91–1.13); PM_10_ 1.10 (0.94–1.30); SO_2_ 1.06 (0.92–1.23)
Corinaldesi et al. [23]	PM_10_	PM_10_ monitoring stations	ICD‐9 code 446.1 as primary discharge diagnosis	Not available	Correlation coefficients: Monthly PM_10_: *R* = 0.20; *U* = 0.20. Annual PM_10_: *R* = −0.25; *U* = −0.32
Lin et al. [29]	PM_10_, SO_2_, NO_2_	Environmental monitoring center	KD diagnostic criteria, including incomplete KD	Mean (SD): PM_10_ 92.9 (54.5) µg/m^3^; SO_2_ 46.0 (26.7) µg/m^3^; NO_2_ 62.3 (23.3) µg/m^3^	Percent increase per 10 µg/m^3^: PM_2.5_ 1.48 (−1.07 to 1.89); SO_2_ 2.11 (−2.70 to 4.41); NO_2_ 3.27 (−0.51 to 7.01), lag day 2
Kuo et al. [27]	PSI, CO, O_3_, NO*_x_*, NO, NO_2_	Air‐quality monitoring stations	ICD‐9‐CM 446.1 and IVIG treatment	Pregnancy: SO_2_ 4.45 ± 1.49 ppb; CO 0.52 ± 0.11 ppm; O_3_ 28.59 ± 3 ppb; PM_10_ 59.37 ± 13.37 µg/m^3^; PM_2.5_ 51.6 ± 61.98 µg/m^3^; NO*_x_* 25.17 ± 7.01 ppb; NO 6.28 ± 2.62 ppb; NO_2_ 18.89 ± 4.65 ppb. Early life: SO_2_ 4.40 ± 1.45 ppb; CO 0.50 ± 0.10 ppm; O_3_ 28.72 ± 2.93 ppb; PM_10_ 58.90 ± 14.84 µg/m^3^; PM_2.5_ 44.91 ± 95.42 µg/m^3^; NO*_x_* 24.47 ± 8.25 ppb; NO 6.06 ± 2.65 ppb; NO_2_ 18.41 ± 5.89 ppb	Pregnancy: CO 1.81 (1.34–2.46); O_3_ 0.99 (0.98–1.00); NO*_x_* 1.01 (1.01–1.02); NO 1.03 (1.02–1.04); NO_2_ 1.01 (1.01–1.02). Postnatal: CO 1.64 (1.18–2.27); O_3_ 1.00 (0.99–1.01); NO*_x_* 1.01 (1.00–1.01); NO 1.02 (1.01–1.03); NO_2_ 1.01 (1.00–1.02)
Kim et al. [26]	PM_2.5_	Prediction modeling	ICD‐10 codes and prescription records	Mean (SD): 28.2 ± 2.9 µg/m^3^	HR per 5 µg/m^3^ increase: 1.21 (1.05–1.39)
Zhu et al. [31]	O_3_	Air‐quality monitoring stations	ICD‐9 code 446.1 or ICD‐10 code M30.3	Mean (SD): O_3_ 87.2 ± 38.2 µg/m^3^; SO_2_ 12.9 ± 9.3 µg/m^3^; NO_2_ 40.7 ± 20.2 µg/m^3^; CO 0.8 ± 0.3 mg/m^3^; PM_2.5_ 43.5 ± 26.6 µg/m^3^; PM_10_ 63.4 ± 39.2 µg/m^3^	Percent change per IQR increase in O_3_: 16.2% (3.6%–30.3%) at lag 0 days; IQR = 52.32 µg/m^3^
Buteau et al. [22]	Ambient PM_2.5_, ambient NO_2_; industrial PM_2.5_, NO_2_, SO_2_	Satellite imaging + geographically weighted regression for PM_2.5_; land‐use regression for NO_2_; dispersion modeling for industrial pollutants	Population‐based health administrative data; ICD‐10 M30.3	Ambient: PM_2.5_ 6.8 µg/m^3^ (IQR 3.8); NO_2_ 11.2 µg/m^3^ (IQR 11.9). Industrial: PM_2.5_ 0.19 µg/m^3^ (IQR 0.13); NO_2_ 1.1 µg/m^3^ (IQR 1.1); SO_2_ 2.3 µg/m^3^ (IQR 1.7)	HR per IQR, prenatal exposure: ambient PM_2.5_ 1.09 (0.84–1.42); ambient NO_2_ 1.03 (0.83–1.28); industrial PM_2.5_ 0.91 (0.83–1.00); industrial NO_2_ 0.97 (0.84–1.12); industrial SO_2_ 1.23 (1.06–1.42)
Fujii et al. [24]	PM_2.5_, NO, NO_2_, SO_2_	Air‐quality monitoring stations	Diagnostic criteria for KD	Mean NO_2_ 14.3 µg/m^3^; mean SO_2_ 11.2 µg/m^3^	Percent increase per 1 µg/m^3^: NO 3.94% (0.04%–7.98%) at lag day 1; NO_2_ −1.32% (−3.08% to 0.48%) at lag day 1; SO_2_ 3.60% (1.12%–6.14%) at lag day 0
Yoneda et al. [30]	PM_2.5_, NO, NO_2_, SO_2_	National Institute for Environmental Studies	Children <5 years diagnosed with KD using ICD‐10 M30.3	Before COVID‐19: PM_2.5_ 11.4 (9.2–13.9) µg/m^3^; NO 2.47 (1.22–4.52) ppb; NO_2_ 7.8 (5.0–11.4) ppb; SO_2_ 1.27 (0.79–1.94) ppb. During COVID‐19: PM_2.5_ 8.5 (6.9–10.3) µg/m^3^; NO 1.72 (0.88–3.07) ppb; NO_2_ 6.2 (4.0–9.2) ppb; SO_2_ 0.83 (0.47–1.24) ppb	Before COVID‐19: PM_2.5_ IRR 1.03 (1.01–1.06); NO 0.99 (0.97–1.01); NO_2_ 1.01 (0.99–1.03); SO_2_ 1.00 (0.96–1.04). During COVID‐19: PM_2.5_ 1.10 (1.04–1.17); NO 0.90 (0.84–0.95); NO_2_ 1.07 (1.02–1.12); SO_2_ 0.94 (0.85–1.04)

Several included studies suggested that meteorological conditions may contribute to the temporal pattern of KD occurrence. Fujii et al. reported an association between preceding hot temperatures and KD incidence, whereas Lin et al. observed a J‐shaped relationship between daily temperature fluctuations and KD incidence. Corinaldesi et al. reported that KD cases occurred predominantly during late autumn and early spring and identified local wind regimes as potential determinants of outbreak timing. These findings suggest that seasonality and meteorological conditions may influence KD occurrence independently of, or in interaction with, air pollution.

### 3.4 PM_2.5_

Evidence for an association between PM_2.5_ exposure and KD was inconsistent across the included studies. Zeft et al. reported no significant association between PM_2.5_ and KD, with an OR of 0.995 (95% CI, 0.868–1.141) per 10 µg/m^3^ increase based on a 14‐day moving average and 0.980 (95% CI, 0.915–1.050) using a 2‐day moving average. In contrast, Yorifuji et al. reported a positive association between prenatal suspended particulate matter exposure and KD hospitalization, with an OR of 1.13 (95% CI, 0.79–1.61) per 10 µg/m^3^ increase, whereas exposure ≥25 µg/m^3^ was associated with an OR of 1.59 (95% CI, 1.06–2.38). The corresponding postnatal estimates were 1.11 (95% CI, 0.73–1.68) per 10 µg/m^3^ increase and 1.41 (95% CI, 0.82–2.41) for exposure ≥25 µg/m^3^. Kwon et al. similarly found no significant association between PM_2.5_ and KD (OR, 1.001; 95% CI, 0.978–1.025 per IQR increase), whereas Oh et al. reported an OR of 0.93 (95% CI, 0.82–1.05) per 10‐unit increase over a 0–14‐day lag period. In contrast, Kim et al. reported a significant positive association between long‐term PM_2.5_ exposure and KD, with an HR of 1.21 (95% CI, 1.05–1.39) per 5 µg/m^3^ increase. Buteau et al. found no significant association for ambient prenatal PM_2.5_ exposure (HR, 1.09; 95% CI, 0.84–1.42 per IQR), although industrial PM_2.5_ exposure showed a borderline association (HR, 0.91; 95% CI, 0.83–1.00). Yoneda et al. reported significant associations between PM_2.5_ and KD incidence both before the COVID‐19 pandemic (IRR, 1.03; 95% CI, 1.01–1.06) and during the pandemic (IRR, 1.10; 95% CI, 1.04–1.17). Overall, the individual‐study findings suggest that the association between PM_2.5_ and KD may depend on exposure timing, duration, and study period rather than representing a uniform effect.

### 3.5 PM_10_

The findings for PM_10_ were also heterogeneous. Kwon et al. reported no significant association between PM_10_ and KD (OR, 0.994; 95% CI, 0.973–1.016 per IQR increase), while Jung et al. reported an OR of 1.10 (95% CI, 0.94–1.30), which was not statistically significant. Corinaldesi et al. found weak correlations between PM_10_ and KD occurrence, with monthly correlation coefficients of 0.20 for both crude and adjusted analyses and annual correlations of −0.25 and −0.32, respectively. Lin et al. reported a percentage increase in KD incidence of 1.48 (95% CI, −1.07 to 1.89) per 10 µg/m^3^ increase in PM_2.5_ at lag day 2; however, this study primarily evaluated PM_10_, SO_2_, and NO_2_ exposure. Buteau et al. did not report a specific ambient PM_10_ association in the available estimates. Overall, the available evidence does not demonstrate a consistent association between PM_10_ exposure and KD.

### 3.6 SO_2_

The evidence for sulfur dioxide (SO_2_) was similarly inconsistent. Kwon et al. found no significant association between SO_2_ and KD (OR, 1.003; 95% CI, 0.972–1.035 per IQR increase), whereas Jung et al. reported an OR of 1.06 (95% CI, 0.92–1.23). Lin et al. reported a 2.11% increase in KD incidence per 10 µg/m^3^ increase in SO_2_ at lag day 2 (95% CI, −2.70 to 4.41), although the CI included the null. In contrast, Fujii et al. reported a significant positive association at lag day 0, with a 3.60% increase in KD incidence (95% CI, 1.12–6.14) per 1 µg/m^3^ increase in SO_2_. Buteau et al. also reported a significant association between prenatal industrial SO_2_ exposure and KD (HR, 1.23; 95% CI, 1.06–1.42 per IQR increase), whereas the ambient pollutant estimates were not significant. Yoneda et al. found no significant association before the COVID‐19 pandemic (IRR, 1.00; 95% CI, 0.96–1.04) or during the pandemic (IRR, 0.94; 95% CI, 0.85–1.04). These findings suggest that any association between SO_2_ and KD may be sensitive to exposure timing, source of exposure, and geographic context.

### 3.7 Pollutant‐Specific Associations Between Air Pollution and KD

#### 3.7.1 NO_2_

Individual studies also produced mixed results for nitrogen dioxide (NO_2_). Kwon et al. reported no significant association (OR, 0.995; 95% CI, 0.962–1.028 per IQR increase), as did Jung et al. (OR, 1.08; 95% CI, 0.89–1.30). Lin et al. observed a 3.27% increase in KD incidence per 10 µg/m^3^ increase in NO_2_ at lag day 2, although the CI crossed the null (95% CI, −0.51 to 7.01). Fujii et al. similarly found no significant association at lag day 1 (−1.32%; 95% CI, −3.08 to 0.48). Buteau et al. reported no significant association for prenatal ambient NO_2_ exposure (HR, 1.03; 95% CI, 0.83–1.28) or industrial exposure (HR, 0.97; 95% CI, 0.84–1.12). However, Kuo et al. reported significant associations for prenatal NO_2_ exposure (OR, 1.01; 95% CI, 1.01–1.02) and postnatal exposure (OR, 1.01; 95% CI, 1.00–1.02). Yoneda et al. found no significant association before the COVID‐19 pandemic (IRR, 1.01; 95% CI, 0.99–1.03) but reported a significant association during the pandemic (IRR, 1.07; 95% CI, 1.02–1.12). Thus, the evidence for NO_2_ remains heterogeneous, with some positive associations emerging in specific exposure periods and study populations.

#### 3.7.2 O_3_

Evidence concerning ozone (O_3_) suggests a possible short‐term association in some settings but not consistently across studies. Kwon et al. found no significant association between O_3_ and KD (OR, 1.001; 95% CI, 0.960–1.043 per IQR increase). In contrast, Jung et al. reported a significant association, with an OR of 1.21 (95% CI, 1.01–1.44) per IQR increase. Zhu et al. also observed a significant short‐term association, with a 16.2% increase in KD onset risk (95% CI, 3.6%–30.3%) per IQR increase in O_3_ concentration at lag 0, whereas associations at subsequent lag days were not significant. Kuo et al. found no significant association for prenatal O_3_ exposure (OR, 0.99; 95% CI, 0.98–1.00) or postnatal exposure (OR, 1.00; 95% CI, 0.99–1.01). Taken together, these findings suggest that O_3_ may have a short‐term or lag‐specific relationship with KD in certain populations, although the evidence is not consistent across studies.

#### 3.7.3 CO

The findings for carbon monoxide (CO) were also mixed. Kwon et al. reported no significant association between CO and KD (OR, 1.010; 95% CI, 0.981–1.040 per IQR increase), whereas Jung et al. similarly found no significant association (OR, 1.01; 95% CI, 0.91–1.13). In contrast, Kuo et al. reported significant associations between CO exposure and KD during both pregnancy (OR, 1.81; 95% CI, 1.34–2.46) and the postnatal period (OR, 1.64; 95% CI, 1.18–2.27). These findings suggest that the relationship between CO and KD may differ according to the developmental period of exposure. However, given the limited number of studies contributing CO‐specific estimates, these findings should be interpreted cautiously.

### 3.8 Meta‐Analysis

The pooled analyses did not demonstrate a statistically significant association between a 10 µg/m^3^ increase in any of the evaluated air pollutants and the occurrence of KD. The summary estimates were RR 1.00 (95% CI, 0.985–1.016) for PM_2.5_, RR 1.00 (95% CI, 0.983–1.022) for PM_10_, RR 1.008 (95% CI, 0.966–1.051) for SO_2_, RR 0.99 (95% CI, 0.989–1.009) for NO_2_, and RR 1.013 (95% CI, 0.981–1.046) for O_3_. The pooled estimate for CO was also reported as RR 1.013 (95% CI, 0.981–1.046). Overall, these findings indicate that the available evidence did not demonstrate a statistically significant change in KD risk associated with a standardized 10 µg/m^3^ increase in individual pollutant concentrations (Figures 2 and 3).

**FIGURE 2:**
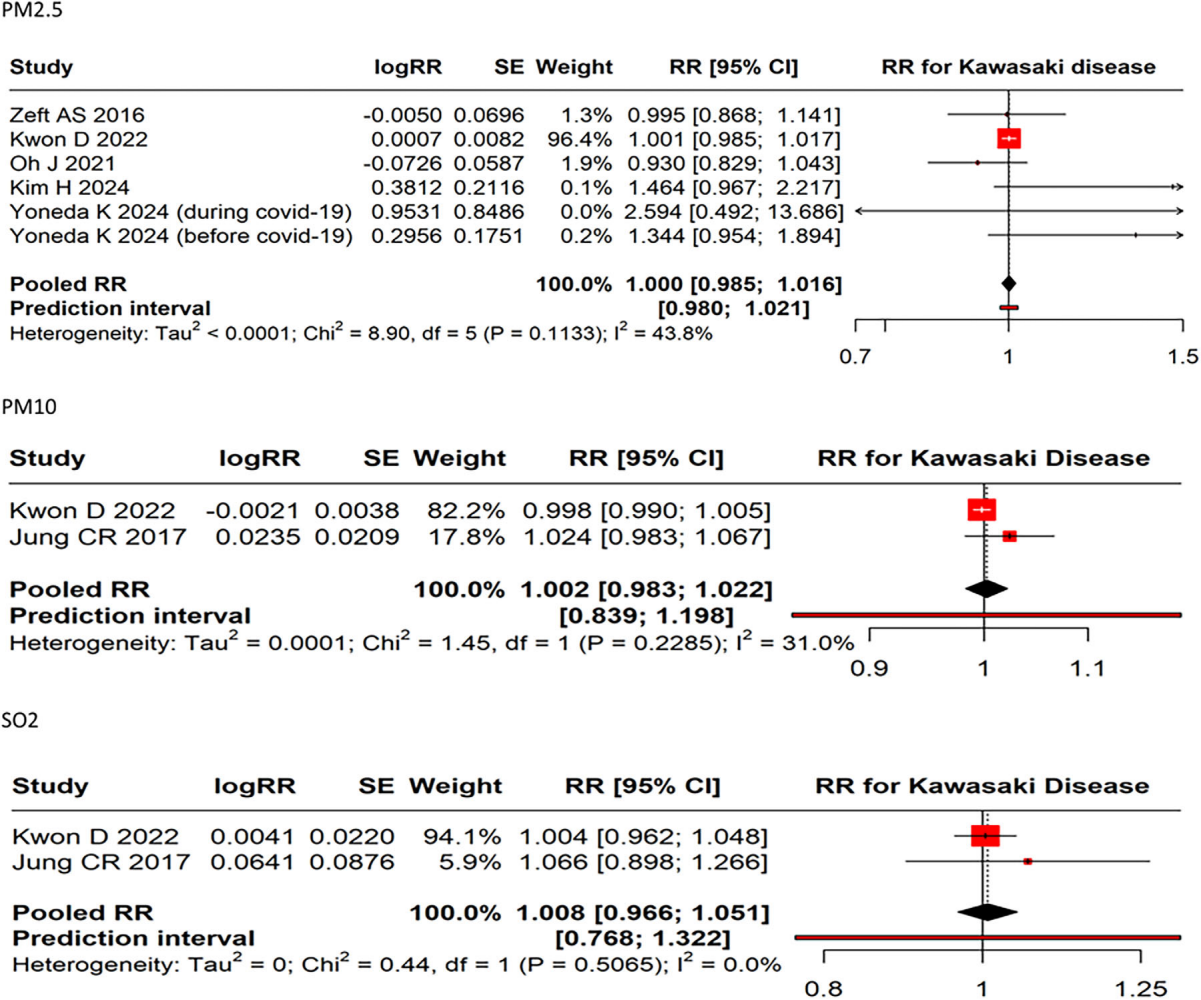
Forest plots for the pooled associations of air pollutants exposure and Kawasaki disease. CI, confidence interval.

**FIGURE 3:**
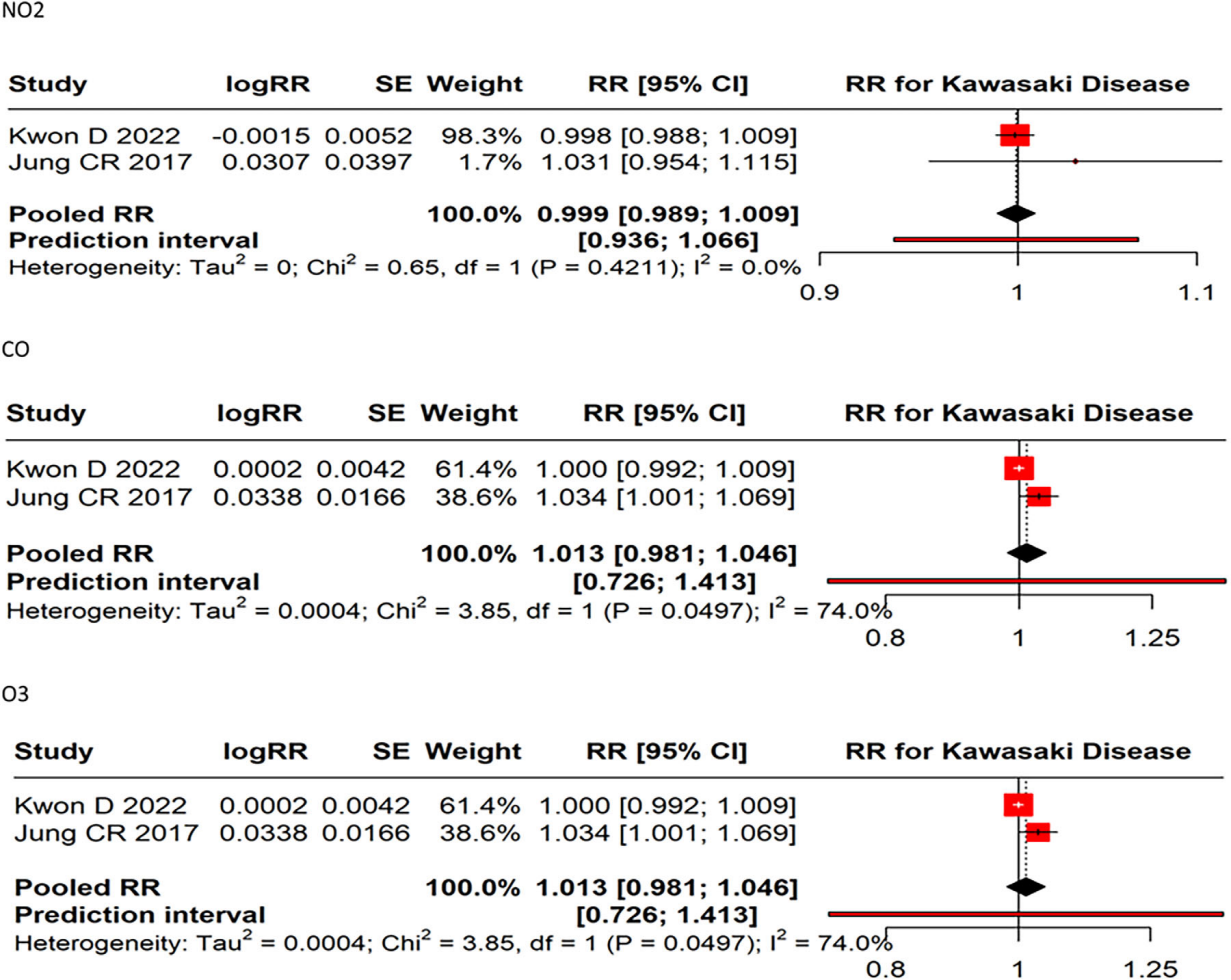
Forest plots for the pooled associations of air pollutants exposure and Kawasaki disease. CI, confidence interval.

For PM_2.5_, between‐study heterogeneity was moderate, with an *I* ^2^ value of 43.8%. Assessment of small‐study effects using Egger's test did not provide evidence of significant publication bias (*p* = 0.2703), supporting the stability of the available evidence for this pollutant.

### 3.9 Sensitivity Analysis

The robustness of the PM_2.5_ meta‐analysis was examined using a leave‐one‐out procedure. Sequential exclusion of individual studies produced no meaningful alteration in the pooled estimate, with the summary effect remaining approximately RR 1.00 (95% CI, 0.985–1.016) (Figure S1). These findings indicate that no single study had a substantial influence on the overall PM_2.5_ estimate and support the stability of the primary analysis.

## 4 Discussion

This systematic review and meta‐analysis examined the available epidemiological evidence on the relationship between ambient air pollution and KD. Across the pooled analyses, a standardized 10 µg/m^3^ increase in PM_2.5_, PM_10_, SO_2_, NO_2_, O_3_, or CO was not associated with a statistically significant increase in KD risk. Nevertheless, the findings across individual studies were heterogeneous, with some studies reporting positive associations for specific pollutants, exposure periods, or lag structures. Taken together, these findings suggest that the relationship between ambient air pollution and KD may be more complex than a uniform pollutant‐related effect and may depend on the type of pollutant, timing and duration of exposure, geographical setting, and accompanying meteorological conditions.

The findings from this meta‐analysis diverge in some respects from earlier research. A study by Yorifuji et al. found that prenatal exposure to suspended particulate matter ≥25 µg/m^3^ was associated with increased KD hospitalization risk [19]. Similarly, Kim et al. noted higher odds of KD with 5 µg/m^3^ increase in the concentrations of PM_2.5_ [26]. In contrast, our analysis did not find a statistically significant relationship between PM_2.5_ exposure and KD risk, indicating that regional variations and methodological differences might account for these inconsistencies in the result.

Several other studies have indicated that other pollutants, such as ozone and nitrogen oxides, could be implicated in KD onset, providing a contrast to our findings [24, 31]. Zhu et al.’s finding of a 16.2% increased risk of KD onset with increased ozone exposure on the day of diagnosis highlights the potential importance of short‐term environmental triggers [31]. Similarly, Fujii et al. reported that pollutants like NO and SO_2_ at elevated concentrations have positive correlation with incidence of KD [24]. However, Lin et al. noted in their study in Shanghai that the effect of air pollutants like SO_2_ and NO_2_ was not significantly associated with KD onset [29]. Jung et al. noted 21% increase in the odds of hospitalization for KD with IQR increase in the concentration of O_3_ [25]. They suggested that ozone exposure, particularly during warmer months, may serve as an acute trigger for KD onset, particularly in susceptible populations such as children because they spend more time outdoors in the summers, thereby increasing their individual exposure levels to ozone [25]. However, Kuo et al. and Kwon et al. observed no associations between ozone and KD in their study in Taiwan [27, 28]. This discrepancy may arise due to differences in geographical settings, pollutant concentrations, and exposure patterns across studies.

Recent systematic reviews and meta‐analyses have further examined the relationship between ambient air pollution and KD, highlighting the evolving and heterogeneous nature of this evidence base. A 2025 systematic review and meta‐analysis by Yang et al., which included 13 studies, reported significant associations for short‐term postnatal exposure to PM_2.5_ and PM_10_ and long‐term postnatal exposure to PM_2.5_, as well as associations for several gaseous pollutants across specific prenatal and postnatal exposure periods [32]. These findings differ from the overall null associations observed in our pollutant‐specific analyses. The discrepancy may reflect differences in exposure windows, lag structures, outcome definitions, study populations, statistical models, and the handling of prenatal versus postnatal exposure. In particular, combining estimates across substantially different exposure periods may obscure associations that are restricted to specific developmental or temporal windows.

A broader 2026 scoping review of outdoor environmental stressors similarly concluded that evidence for short‐term air pollution effects on KD remains inconsistent, whereas associations with long‐term or prenatal particulate matter exposure appear more consistent [33]. The same review emphasized the potential contribution of meteorological variables, including temperature and wind patterns, and highlighted substantial methodological heterogeneity across studies. These observations support the interpretation that air pollution may not exert a uniform effect on KD risk but may instead interact with exposure timing, meteorological conditions, geographic characteristics, and individual susceptibility.

Accordingly, the absence of a statistically significant overall association in the present analysis should not be interpreted as evidence that air pollution has no role in KD. Rather, the findings suggest that any association may be context‐dependent and may be more apparent for particular pollutants, exposure windows, or environmental settings. Future studies should therefore prioritize standardized exposure definitions, prespecified lag periods, consistent adjustment for meteorological variables, and separate analyses of prenatal, early‐life, and short‐term exposures.

Beyond air pollution, other environmental factors, such as temperature and climate, have been shown to affect KD incidence. For instance, Lin et al. found that temperature, rather than air pollutants, had a significant impact on KD incidence in Shanghai, suggesting that exposure to extreme heat could increase the risk of KD [29]. Similarly, Corinaldesi et al. suggested that wind regimes, rather than pollution alone, influenced the timing of KD outbreaks in Emilia‐Romagna, Italy [23]. These findings emphasize the multifactorial nature of KD and highlight the potential for climatic factors to interact with air pollution to influence KD risk. The interaction between air pollution and weather patterns is highly intricate, and this complexity might explain why the pooled relative risks for various air pollutants in the studies reviewed did not yield consistent results.

Although pollutant‐specific associations were examined separately, these estimates should not be interpreted as independent causal contributions of individual pollutants because exposure to ambient pollutants is highly correlated and the included studies differed in exposure windows, lag structures, exposure assessment methods, and covariate adjustment.

This meta‐analysis provides an updated quantitative assessment of the association between individual ambient air pollutants and KD and further highlights substantial heterogeneity related to exposure timing, geographic setting, and meteorological conditions.

The variability in study findings highlights the importance of considering region‐specific factors, such as environmental conditions and pollutant profiles. Different geographic regions experience unique patterns of air pollution, temperature, humidity, and wind, all of which can modulate the concentration and dispersion of pollutants. These regional and climatic factors complicate our ability to draw universal conclusions about the relationship between air pollution and KD. This variability highlights the need for more regionally focused studies that can better elucidate these complex relationships.

Another limitation is the relatively small number of studies included in the meta‐analysis, which may have limited the statistical power to detect significant associations. Larger studies, including those covering diverse regions and population groups, would provide a more robust understanding of how air pollutants influence KD risk. Another limitation is the inconsistent adjustment for meteorological factors across studies. Temperature, humidity, wind patterns, and seasonality may influence both pollutant concentrations and KD incidence, creating potential confounding or effect modification. Because these variables were not uniformly measured or incorporated into the statistical models, the independent contribution of individual pollutants cannot be established. In addition, the included studies used heterogeneous exposure windows, including prenatal, early‐life, short‐term, and lagged exposures. Pooling estimates across these distinct exposure periods may attenuate associations that are restricted to specific temporal or developmental windows. Furthermore, most studies in this review focused on outdoor air pollution, whereas indoor air pollution, often more prevalent in developing countries, has been largely overlooked. Children in these regions may be particularly vulnerable to indoor pollutants, making this an important area for future research. Investigating how indoor air pollution affects KD risk could yield valuable insights into the broader environmental determinants of the disease.

Given the multifactorial nature of KD (air pollution, temperature, and other climatic factors), interventions aimed at reducing air pollution alone may not be sufficient to prevent KD. Public health strategies should address a broader range of environmental factors, including temperature regulation and reducing exposure to pollutants, especially for vulnerable groups like children to mitigate KD risk. Further research is needed to explore the relationship between air pollution and KD, with region‐specific studies that consider local pollutant profiles, climate, and population vulnerability. Additionally, future research should investigate how air pollution (outdoor and indoor), temperature, and other climatic factors interact in contributing to KD risk.

## 5 Conclusion

The evidence linking ambient air pollution to KD remains inconclusive. Although the overall pollutant‐specific pooled estimates did not demonstrate statistically significant associations, individual studies suggest that associations may vary according to pollutant type, exposure timing, geographic setting, and meteorological conditions. Future studies should use standardized exposure windows and consistently account for temperature, seasonality, humidity, and other meteorological factors to clarify potential context‐specific associations between air pollution and KD.

## Author Contributions

Amogh Verma, Pratima Pandey, Asif Jan, and Daniel Dubwa conceptualized the study. Amogh Verma and Daniel Dubwa developed the methodology. Ranjana Sah, Asif Jan, and Daniel Dubwa handled software. Ranjana Sah curated the data. Amogh Verma, Ranjana Sah, Pratima Pandey, Asif Jan, and Daniel Dubwa performed investigation and validation. Ranjana Sah and Asif Jan conducted formal analysis. Asif Jan supervised the study. Daniel Dubwa acquired funding. Pratima Pandey, Asif Jan, and Daniel Dubwa generated visualizations. Asif Jan and Daniel Dubwa drafted the manuscript. Amogh Verma, Ranjana Sah, Asif Jan, and Daniel Dubwa reviewed and edited the manuscript.

## Funding

The authors have nothing to report.

## Ethics Statement

The authors have nothing to report.

## Conflicts of Interest

The authors declare no conflicts of interest.

## Supporting information




**Supporting File 01:** puh270448‐sup‐0001‐SuppMat.docx

## Data Availability

The data are with the authors and available on request.

## References

[1] R. Scuccimarri , “Kawasaki Disease,” Pediatric Clinics of North America 59, no. 2 (2012): 425–445. 10.1016/j.pcl.2012.03.009 22560578

[2] Y. Onouchi , “Genetics of Kawasaki Disease: What We Know and Don't Know,” Circulation Journal 76, no. 7 (2012): 1581–1586. 10.1253/circj.cj-12-0568 22789975

[3] J. C. Burns , L. Herzog , O. Fabri , et al., “Seasonality of Kawasaki Disease: A Global Perspective,” PLoS ONE 8, no. 9 (2013): e74529. 10.1371/journal.pone.0074529 PMC3776809 24058585

[4] X. Rodó , J. Ballester , D. Cayan , et al., “Association of Kawasaki Disease With Tropospheric Wind Patterns,” Scientific Reports 1, no. 1 (2011): 152. 10.1038/srep00152 PMC3240972 22355668

[5] W. Checkley , J. Guzman‐Cottrill , L. Epstein , N. Innocentini , J. Patz , and S. Shulman , “Short‐Term Weather Variability in Chicago and Hospitalizations for Kawasaki Disease,” Epidemiology 20, no. 2 (2009): 194–201. 10.1097/EDE.0b013e3181961a9b 19129731

[6] H. Jorquera , A. Borzutzky , R. Hoyos‐Bachiloglu , and A. García , “Association of Kawasaki Disease With Tropospheric Winds in Central Chile: Is Wind‐borne Desert Dust a Risk Factor?,” Environment International 78 (2015): 32–38. 10.1016/j.envint.2015.02.007 25743034

[7] X. Rodó , R. Curcoll , M. Robinson , et al., “Tropospheric Winds From Northeastern China Carry the Etiologic Agent of Kawasaki Disease From Its Source to Japan,” Proceedings of the National Academy of Sciences 111, no. 22 (2014): 7952–7957. 10.1073/pnas.1400380111 PMC4050536 24843117

[8] C.‐N. Zhao , Z. Xu , G.‐C. Wu , et al., “Emerging Role of Air Pollution in Autoimmune Diseases,” Autoimmunity Reviews 18, no. 6 (2019): 607–614. 10.1016/j.autrev.2018.12.010 30959217

[9] S. C. L. Farhat , C. A. Silva , M. A. M. Orione , L. M. A. Campos , A. M. E. Sallum , and A. L. F. Braga , “Air Pollution in Autoimmune Rheumatic Diseases: A Review,” Autoimmunity Reviews 11, no. 1 (2011): 14–21. 10.1016/j.autrev.2011.06.008 21763467

[10] C. A. Pope III , J. B. Muhlestein , H. T. May , D. G. Renlund , J. L. Anderson , and B. D. Horne , “Ischemic Heart Disease Events Triggered by Short‐Term Exposure to Fine Particulate Air Pollution,” Circulation 114, no. 23 (2006): 2443–2448. 10.1161/CIRCULATIONAHA.106.636977 17101851

[11] R. Rückerl , A. Schneider , S. Breitner , J. Cyrys , and A. Peters , “Health Effects of Particulate Air Pollution: A Review of Epidemiological Evidence,” Inhalation Toxicology 23, no. 10 (2011): 555–592. 10.3109/08958378.2011.593587 21864219

[12] W. J. Gauderman , E. Avol , F. Gilliland , et al., “The Effect of Air Pollution on Lung Development From 10 to 18 Years of Age,” New England Journal of Medicine 351, no. 11 (2004): 1057–1067. 10.1056/NEJMoa040610 15356303

[13] A. Barraza‐Villarreal , J. Sunyer , L. Hernandez‐Cadena , et al., “Air Pollution, Airway Inflammation, and Lung Function in a Cohort Study of Mexico City Schoolchildren,” Environmental Health Perspectives 116, no. 6 (2008): 832–838. 10.1289/ehp.10926 PMC2430242 18560490

[14] S. Naoe , K. Takahashi , H. Masuda , and T. N. Kawasaki , “Disease With Particular Emphasis on Arterial Lesions,” Acta Pathologica Japonica 41, no. 11 (1991): 785–797. 10.1111/j.1440-1827.1991.tb01620.x 1785339

[15] Y. Lee , D. J. Schulte , K. Shimada , et al., “Interleukin‐1β is Crucial for the Induction of Coronary Artery Inflammation in a Mouse Model of Kawasaki Disease,” Circulation 125, no. 12 (2012): 1542–1550. 10.1161/CIRCULATIONAHA.111.072769 PMC3337219 22361326

[16] L. T. Hoang , C. Shimizu , L. Ling , et al., “Global Gene Expression Profiling Identifies New Therapeutic Targets in Acute Kawasaki Disease,” Genome Medicine 6 (2014): 541. 10.1186/s13073-014-0102-6 PMC4279699 25614765

[17] R. N. Bauer , D. Diaz‐Sanchez , and I. Jaspers , “Effects of Air Pollutants on Innate Immunity: The Role of Toll‐Like Receptors and Nucleotide‐binding Oligomerization Domain‐Like Receptors,” Journal of Allergy and Clinical Immunology 129, no. 1 (2012): 14–24. 10.1016/j.jaci.2011.11.004 PMC4341993 22196521

[18] H. Sulthana , J. Jays , and B. Prakash Kumar , “The Convergence of Statistical Design and Computational Intelligence: A Systematic Review of DOE‐Driven AI/ML in Analytical Method Development and Validation,” Accreditation and Quality Assurance 31, no. 3 (2026): 177–190.

[19] T. Yorifuji , H. Tsukahara , S. Kashima , and H. Doi , “Intrauterine and Early Postnatal Exposure to Particulate Air Pollution and Kawasaki Disease: A Nationwide Longitudinal Survey in Japan,” Journal of Pediatrics 193 (2018): 147–154.e2. 10.1016/j.jpeds.2017.10.012 29212623

[20] A. S. Zeft , J. C. Burns , R. S. Yeung , et al., “Kawasaki Disease and Exposure to Fine Particulate Air Pollution,” Journal of Pediatrics 177 (2016): 179–183.e1. 10.1016/j.jpeds.2016.06.061 27496266

[21] J. Oh , J. H. Lee , E. Kim , S. Kim , H. S. Kim , and E. Ha , “Is Short‐Term Exposure to PM_2.5_ Relevant to Childhood Kawasaki Disease?,” International Journal of Environmental Research and Public Health 18, no. 3 (2021): 924.

[22] S. Buteau , S. Belkaibech , M. Bilodeau‐Bertrand , M. Hatzopoulou , A. Smargiassi , and N. Auger , “Association Between Kawasaki Disease and Prenatal Exposure to Ambient and Industrial Air Pollution: A Population‐Based Cohort Study,” Environmental Health Perspectives 128, no. 10 (2020): 107006. 10.1289/EHP6920 PMC7571626 33074736

[23] E. Corinaldesi , V. Pavan , L. Andreozzi , et al., “Environmental Factors and Kawasaki Disease Onset in Emilia‐Romagna, Italy,” International Journal of Environmental Research and Public Health 17, no. 5 (2020): 1529. 10.3390/ijerph17051529 PMC7084934 32120916

[24] F. Fujii , N. Egami , M. Inoue , and H. Koga , “Weather Condition, Air Pollutants, and Epidemics as Factors That Potentially Influence the Development of Kawasaki Disease,” Science of the Total Environment 741 (2020): 140469. 10.1016/j.scitotenv.2020.140469 32610244

[25] C.‐R. Jung , W.‐T. Chen , Y.‐T. Lin , and B.‐F. Hwang , “Ambient Air Pollutant Exposures and Hospitalization for Kawasaki Disease in Taiwan: A Case‐Crossover Study (2000–2010),” Environmental Health Perspectives 125, no. 4 (2017): 670–676. 10.1289/EHP137 PMC5381970 27458717

[26] H. Kim , H. Jang , W. Lee , et al., “Association Between Long‐Term PM_2.5_ Exposure and Risk of Kawasaki Disease in Children: A Nationwide Longitudinal Cohort Study,” Environmental Research 244 (2024): 117823. 10.1016/j.envres.2023.117823 38072109

[27] N.‐C. Kuo , C.‐H. Lin , and M.‐C. Lin , “Prenatal and Early Life Exposure to Air Pollution and the Incidence of Kawasaki Disease,” Scientific Reports 12, no. 1 (2022): 3415. 10.1038/s41598-022-07081-y PMC8888747 35233028

[28] D. Kwon , Y. J. Choe , S. Y. Kim , B. C. Chun , and S. A. Choe , “Ambient Air Pollution and Kawasaki Disease in Korean Children: A Study of the National Health Insurance Claim Data,” Journal of the American Heart Association 11, no. 9 (2022): e024092. 10.1161/JAHA.121.024092 PMC9238605 35475377

[29] Z. Lin , X. Meng , R. Chen , et al., “Ambient Air Pollution, Temperature and Kawasaki Disease in Shanghai, China,” Chemosphere 186 (2017): 817–822. 10.1016/j.chemosphere.2017.08.054 28822259

[30] K. Yoneda , D. Shinjo , N. Takahashi , and K. Fushimi , “Spatiotemporal Analysis of the Association Between Kawasaki Disease Incidence and PM_2.5_ Exposure: A Nationwide Database Study in Japan,” BMJ Paediatrics Open 8, no. 1 (2024): e002887. 10.1136/bmjpo-2024-002887 PMC11428985 39327060

[31] Y. Zhu , R. Chen , C. Liu , et al., “Short‐Term Exposure to Ozone May Trigger the Onset of Kawasaki Disease: An Individual‐Level, Case‐Crossover Study in East China,” Chemosphere 349 (2024): 140828. 10.1016/j.chemosphere.2023.140828 38040257

[32] P. Yang , J. Zhang , K. Zhang , et al., “Prenatal and Postnatal Ambient Air Pollution and Kawasaki Disease: A Systematic Review and Meta‐Analysis,” JACC: Advances 4, no. 4 (2025): 101651. 10.1016/j.jacadv.2025.101651 PMC11937670 40088736

[33] L. Madaniyazi , J. Alpizar , C.‐R. Jung , et al., “Kawasaki Disease and Outdoor Environmental Stressors: A Scoping Review,” Lancet Regional Health—Western Pacific 66 (2026): 101791. 10.1016/j.lanwpc.2025.101791 PMC12805108 41550610

